# Supplementary material for: Gene Editing of the Follicle-Stimulating Hormone Gene to Sterilize Channel Catfish, Ictalurus punctatus, Using a Modified Transcription Activator-like Effector Nuclease Technology with Electroporation
Source: Biology (Basel). 2023 Mar 1;12(3):392. doi: 10.3390/biology12030392 (PMC10044888; doi:10.3390/biology12030392)
Supplement: Supplementary file 1 [file biology-12-00392-s001.zip › biology-2245501-supplementary.pdf]

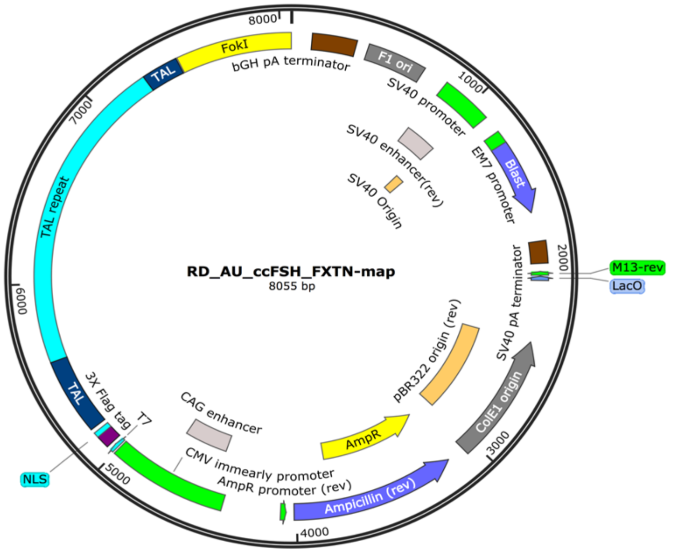

Figure S1. Schematic representation of transcription activator-like effector nucleases (TALENs) plasmid structure targeting follicle-stimulating hormone (*fsh*) gene of channel catfish (*Ictalurus punctatus*). *CMV prmt* cytomegalovirus promoter, *NLS* nuclear localization signal, *BGH pA* bovine growth hormone polyadenylation signal, *AmpR* ampicillin resistance gene.

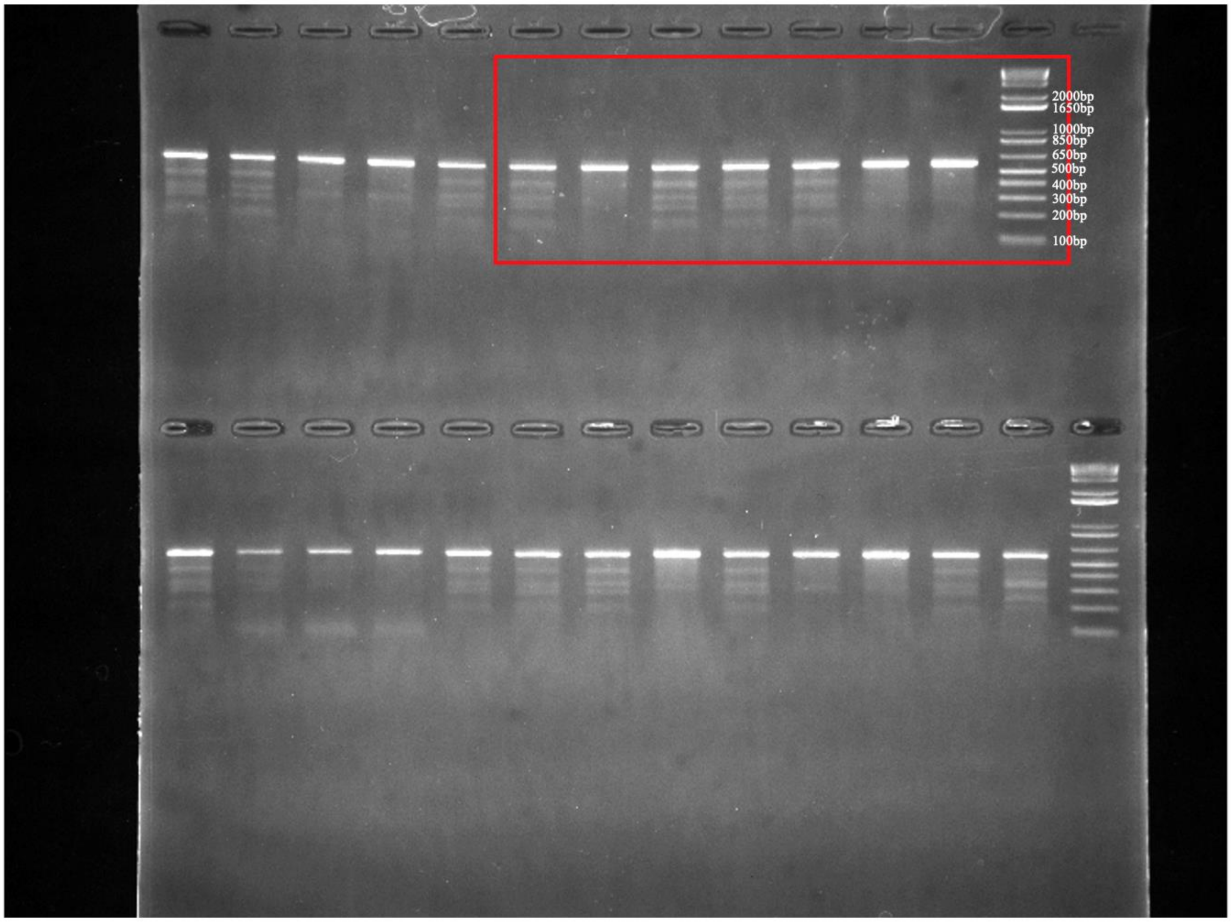

Figure S2. Original images of gels. The result of Surveyor mutation detection in Fig. 1 was shown. Red box represents the cropped area.

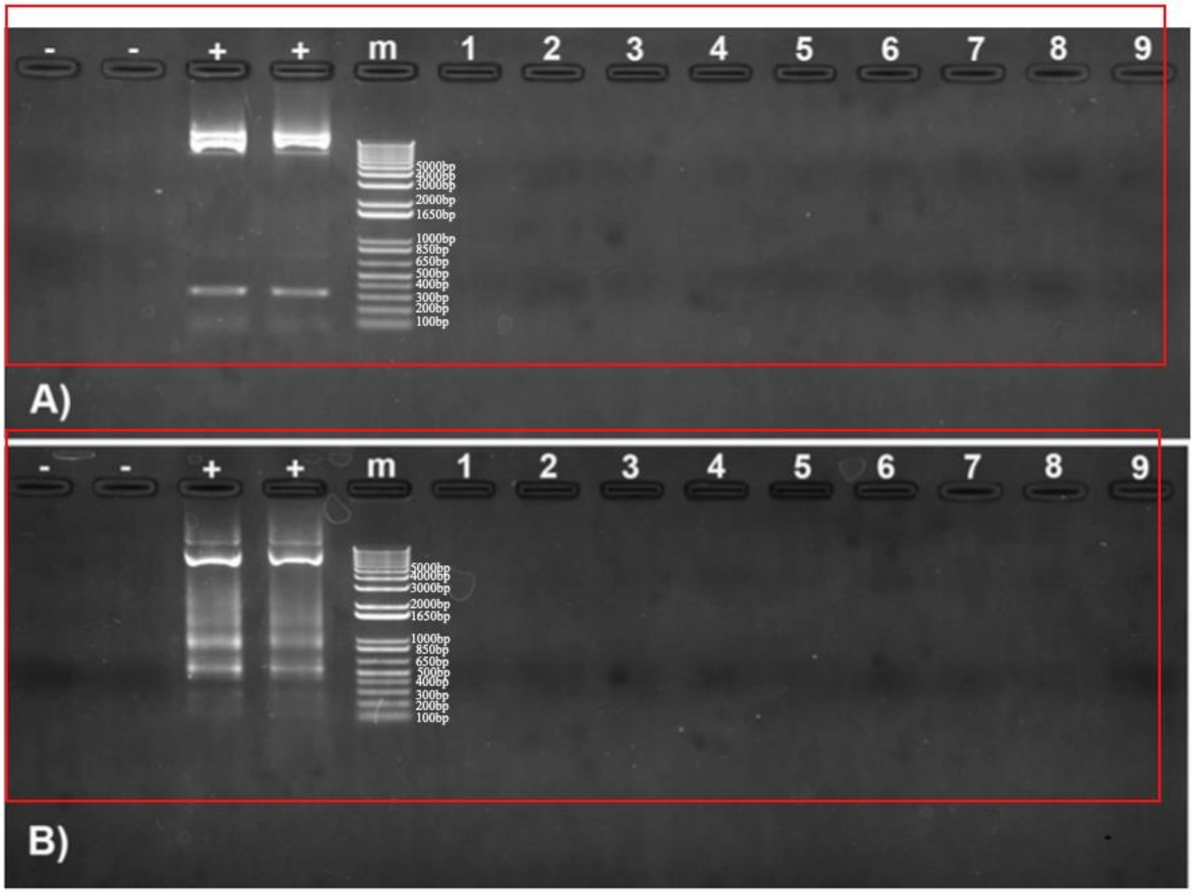

Figure S3. Original images of gels. The result of PCR inspection in Fig. 3 was shown. Red box represents the cropped area.

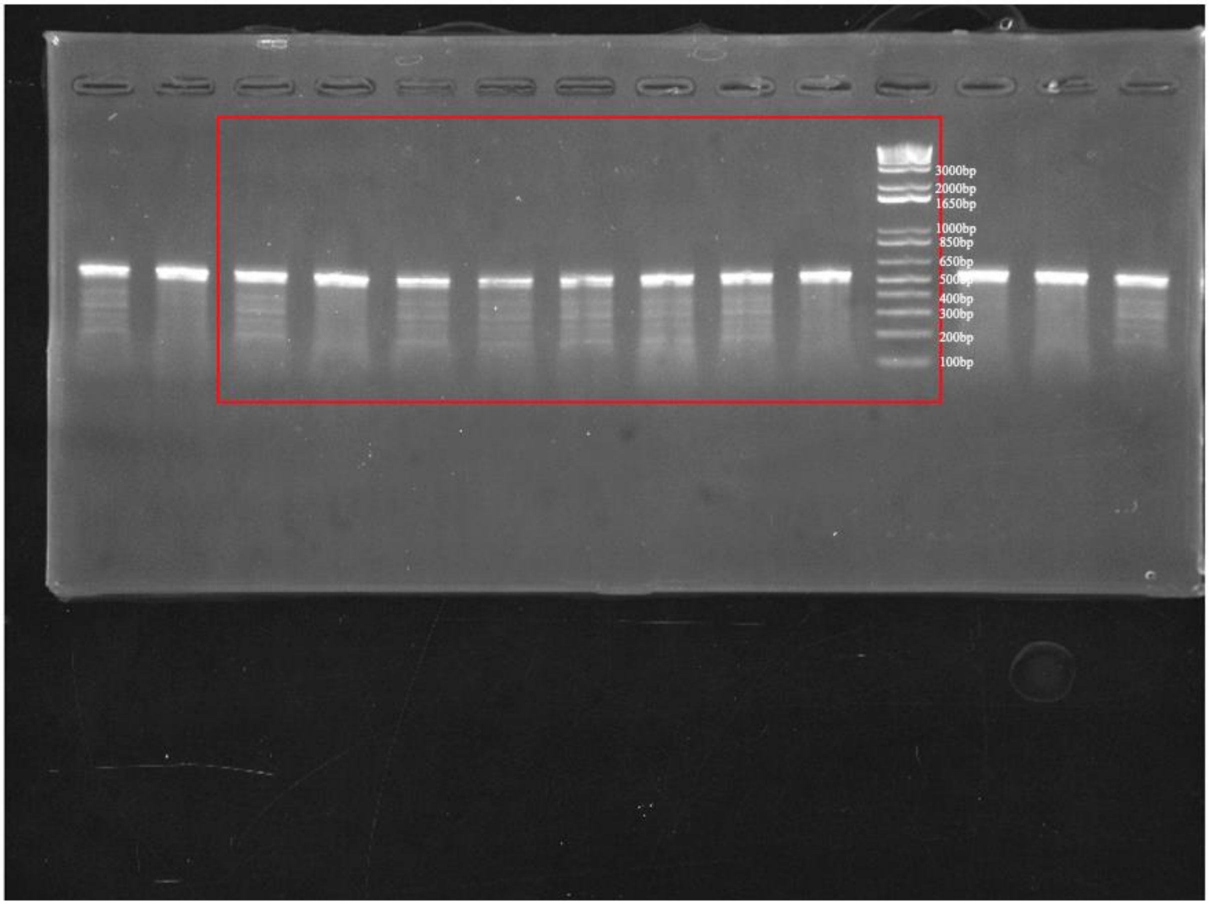

Figure S4. Original images of gels. The result of Surveyor mutation detection in Fig. 4 was shown. Red box represents the cropped area.

Table S1. Primer sequences used for the amplification of follicle-stimulating hormone (*fsh*), cytomegalovirus (CMV) promoter, and transcription activator-like (TAL) repeats regions in channel catfish (*Ictalurus punctatus*).

Targeting site	Sequence	Annealing temperature	Product length (bp)
fsh Forward	5′-CACAACTCCAGCTGTGACAA-3′	60 °C	550
fsh Reverse	5′-CAGAATTCCGTGGCCATTTA-3′		
CMV promoter Forward	5′-AACAACAACGGCGGTAAG-3′	60 °C	114
CMV promoter Reverse	5′-CCCATTATTGTTCGCGATTG-3′		
TAL Repeats Forward	5′-GCATGACGGAGGGAAAC-3′	60 °C	215
TAL Repeats Reverse	5′-CCATTATTGTTCGCGATTGA-3′		
